# Artificial intelligence approaches and mechanisms for big data analytics: a systematic study

**DOI:** 10.7717/peerj-cs.488

**Published:** 2021-04-14

**Authors:** Amir Masoud Rahmani, Elham Azhir, Saqib Ali, Mokhtar Mohammadi, Omed Hassan Ahmed, Marwan Yassin Ghafour, Sarkar Hasan Ahmed, Mehdi Hosseinzadeh

**Affiliations:** 1 Future Technology Research Center, National Yunlin University of Science and Technology, Yunlin, Taiwan; 2 Department of Computer Science, Khazar University, Baku, Azerbaijan; 3 Department of Computer Engineering, Science and Research Branch, Islamic Azad University, Tehran, Iran; 4 Department of Information Systems, College of Economics and Political Science, Sultan Qaboos University, Muscat, Oman; 5 Department of Information Technology, Lebanese French University, Erbil, Kurdistan Region, Iraq; 6 Department of Information Technology, University of Human Development, Sulaymaniyah, Iraq; 7 Department of Computer Science, College of Science, University of Halabja, Halabja, Iraq; 8 Network Department, Sulaimani Polytechnic University, Sulaymaniyah, Iraq; 9 Institute of Research and Development, Duy Tan University, Da Nang, Vietnam; 10 Mental Health Research Center, Psychosocial Health Research Institue, Iran University of Medical Sciences, Tehran, Iran

**Keywords:** Big data, Artificial intelligence, Machine learning, Methods, Systematic literature review

## Abstract

Recent advances in sensor networks and the Internet of Things (IoT) technologies have led to the gathering of an enormous scale of data. The exploration of such huge quantities of data needs more efficient methods with high analysis accuracy. Artificial Intelligence (AI) techniques such as machine learning and evolutionary algorithms able to provide more precise, faster, and scalable outcomes in big data analytics. Despite this interest, as far as we are aware there is not any complete survey of various artificial intelligence techniques for big data analytics. The present survey aims to study the research done on big data analytics using artificial intelligence techniques. The authors select related research papers using the Systematic Literature Review (SLR) method. Four groups are considered to investigate these mechanisms which are machine learning, knowledge-based and reasoning methods, decision-making algorithms, and search methods and optimization theory. A number of articles are investigated within each category. Furthermore, this survey denotes the strengths and weaknesses of the selected AI-driven big data analytics techniques and discusses the related parameters, comparing them in terms of scalability, efficiency, precision, and privacy. Furthermore, a number of important areas are provided to enhance the big data analytics mechanisms in the future.

## Introduction

With the rapid innovations of digital technologies, the volume of digital data is growing fast (Klein, 2017). Consequently, large quantities of data are created from lots of sources such as social networks, smartphones, sensors, etc. Such huge amounts of data that conventional relational databases and analytical techniques are unable to store and process is called Big Data. Development of novel tools and analytical techniques are therefore required to discover patterns from large datasets. Big data is produced quickly from numerous sources in multiple formats. Henceforth, the novel analytical tools should be able to detect correlations between rapidly changing data to better exploit them.

As mentioned, traditional processing techniques have problems coping with a huge amount of data. It’s necessary to develop effective ways for data analysis in big data problems. Various big data frameworks such as Hadoop and Spark have allowed a lot of data to be distributed and analyzed (Oussous et al., 2018). Furthermore, different types of Artificial Intelligence (AI) techniques, such as Machine Learning (ML) and search-based methods were introduced to deliver faster and more precise results for large data analytics. The combination of big data tools and AI techniques has created new opportunities in big data analysis.

There are some literatures reviews on big data analytics techniques. Nevertheless, none of these articles concentrate on the complete and systematic review of the artificial intelligent mechanisms for big data analytics. We have studied and classified the articles in the field of big data analytics using artificial intelligent techniques. The AI-driven big data analytics techniques will be described together with the strengths and weaknesses of every technique. In this survey, the existing research on big data analytics techniques is categorized into four major groups, including machine learning, knowledge-based and reasoning methods, decision-making algorithms, and search methods and optimization theory. This survey makes three main contributions as follows:

• Providing a systematic study related to big data analytics using AI techniques.
• Classifying and reviewing AI-driven big data analytics techniques in four main categories, and specifying their key advantages and disadvantages.
• Discussing open issues to provide new research directions in the big data analysis filed.

The following classification will be discussed in the rest of the paper. The previous studies have been reviewed in “Background and Related Work”. In “Research Selection Method”, we described the process of article selection. The intended taxonomy for the chosen big data analysis studies and the selected studies are reviewed in “AI-driven big Data Analytics Mechanisms”. The investigated studies will be compared in “Results and Comparisons”. Eventually, some open issues and the conclusion are provided in “Open Issues and Challenges” and “Conclusion”, respectively.

## Background and Related Work

In this part, some preliminaries and related works for big data analytics are illustrated.

### Big data definition and characteristics

Huge volumes of data gathered from various sources like sensors, transactional applications, and social media in heterogeneous formats. There are various definitions presented for big data (I.I.J, 2014; Gantz & Reinsel, 2012; Glossary, 2014; Manyika et al., 2011; Chang et al., 2015). Generally, the term Big Data refers to a growing set of data that contain varied formats: structured, unstructured, and semi-structured data. Existing Database Management Systems (DBMSs) are not able to process such a huge volume of heterogeneous data. Therefore, powerful technologies and advanced algorithms are needed for processing big data.

The big data can be described using different V’s such as Volume, Velocity, Variety, Veracity (Furht & Villanustre, 2016).

• Volume: This implies the huge quantities of data produced every second. These huge volumes of data can be processed in big data frameworks.
• Velocity: This denotes the speed of data production and processing to extract valuable insights.
• Variety: This specifies the various format of data such as documents, videos, and logs.
• Veracity: This indicates the data quality factors. That is, it specifies the biases, noise, abnormality etc. in the data.

Nowadays, more V’s and other characteristics such as Visualization, Value, and Volatility have been used to better define big data (Patgiri & Ahmed, 2016).

Management of big data is essential to efficiently manage big data for creating quality data analytics. It includes efficient data collection from different sources, efficient storage using various mechanisms and tools, data cleansing to eliminate the errors and transform the data into a uniform format, and data encoding for security and privacy. The goal of this process is to ensure the availability, management, efficient and secure storage of reliable data.

### Big data analytics

Organizations can extract valuable information and patterns that may affect business through big data analytics (Gandomi & Haider, 2015). Thus, advanced data analysis is needed to identify the relations between features and forecast future observations. Big data analytics refers to techniques applied to achieve insights from huge datasets (Labrinidis & Jagadish, 2012). The big data analytics results can improve decision-making and increase organizational efficiency. Various analytical approaches are developed to extract knowledge from the data, such as:

• **Descriptive analytics** is concerned with analyzing historical data of a business to describe what occurred in the past (Joseph & Johnson, 2013).
• **Predictive analytics** is focused on a variety of statistical modeling and machine learning techniques to predict future possibilities (Waller & Fawcett, 2013).
• **Prescriptive analytics** include descriptive and predictive analytics to recommend the most suitable actions to enhance business practices (Joseph & Johnson, 2013).

Data mining, statistical analysis, machine learning, rule-based systems, neural networks, and etc. are various analytics techniques to make better and faster decisions on big data sets to uncover hidden patterns. Various researches address this field of study by improving the developed techniques, proposing novel methods, or investigating the combination of various algorithms. However, more analytical improvements are required to meet the challenges of big data (Oussous et al., 2018).

### Big data platforms

Batch processing, real-time processing, and interactive analytics are different platforms of big data (Borodo, Shamsuddin & Hasan, 2016). Batch processing platforms perform extensive computations and take time to process data. Apache Hadoop is the most common batch processing platform. It is used due to scalability, cost-effectiveness, flexibility, and fault tolerance in the big data processing. Hadoop Distributed File System (HDFS), Yet Another Resource Negotiator (YARN), and MapReduce distributed programming model are some different modules of the Hadoop platform which operate across the big data value chain; from aggregation, storage, process, and management.

As defined in the previous sections, velocity is another characteristic of big data. It is defined as a continuous, and high-speed data streams that arrive at rapid rates, and requires continuous processing and analysis. Real-time processing platforms are used for fast and efficient analysis of continuous data streams. Apache Spark (Acharjya & Ahmed, 2016) and Storm (Mazumder, 2016) are two common examples of stream processing platforms. Stream processing would be required for various applications such as weather and transport systems.

Interactive analytics platforms enable users to access dataset remotely and perform various operations as needed. Users can connect to a system directly and interact with data. Apache Drill is an example of interactive analytic platforms.

### Related work

A brief overview of the previous survey studies is presented in this part. Here the previous surveys are classified into four main categories include big data management process; big data analytics techniques; big data platforms; and big data analytics applications. We discuss these surveys in the following subsections.

#### Big data management process

The authors in Tsai et al. (2015) reviewed various studies related to the traditional and recent big data analysis. The procedure of Knowledge Discovery in Data mining (KDD) involving input, analysis, and output is considered as the basis for these studies. Various data and big data mining techniques such as clustering and classification are discussed in the analysis step. Moreover, some open issues and future research directions have been suggested to provide efficient methods. However, their survey has not been written in a systematic way, the studies are not compared completely and the recently published articles are not included. Also, they only have focused on the machine learning category of artificial intelligence techniques, and other AI categories such as computational intelligence have not been studied.

Siddiqa et al. (2016) presented a basic overview of various big data management techniques. A detailed taxonomy was presented based on storage, pre-processing, processing, and security. Various articles have been discussed in each category. Furthermore, the features of the proposed methods were described in this paper, and different techniques were compared. Moreover, future works and open challenges have been discussed. However, there is no clear method for article selection.

#### Big data analytics techniques

Athmaja, Hanumanthappa & Kavitha (2017) presented a systematic literature-based review of the big data analytics approaches according to the machine learning mechanisms. However, no categorization is provided for reviewing related studies in the present paper. Moreover, the non-functional features of the studies have not been investigated. The authors do not provide any systematic procedure for gathering the related studies.

Ghani et al. (2019) have reviewed the existing big social media analytics approaches in five classes: artificial neural networks, fuzzy systems, swarm intelligence, evolutionary computation, and deep learning. The authors assessed the reviewed techniques based on their quality metrics. However, there is no systematic procedure to select articles related to this field.

A complete study of big data analysis tools and techniques has been presented by Mittal & Sangwan (2019). The authors focused on studying machine learning techniques for big data analysis. Therefore, three categories are considered for reviewing selected techniques, which include supervised learning, unsupervised learning, and reinforcement learning. Nevertheless, there is no clear method for article selection and the studies have not been evaluated based on quality parameters.

Qiu et al. (2016) presented a brief review of the ML techniques. Some recent learning methods, such as representation learning, deep learning, distributed and parallel learning, transfer learning, active learning, and kernel-based learning are highlighted in this review article. However, they only focused on machine learning techniques and the study reviews few papers in each classification. Also, the article selection procedure is not included in this paper. Moreover, in this paper, no technical comparison has been made in relation to the proposed methods.

Another work provided by Sivarajah et al. (2017) for the big data analysis techniques. The authors categorized these techniques into three main groups, including descriptive, predictive, and prescriptive analytics. However, there are some gaps in analyzing the qualitative parameters, and the study selection process.

#### Big data platforms

Oussous et al. (2018) investigated the impact of big data challenges, and numerous tools for its analysis. The tools used for big data processing are discussed in this article. Also, the challenges of big data analytics are divided into six general categories: big data management, big data cleansing, big data collection, imbalanced big data, big data analytics, and big data machine learning. However, the article selection process is not included. Also, there are no categories in this article based on some factors.

Nicolalde et al. (2018) investigated research efforts directed toward big data processing technologies. The authors discussed some associated challenges, such as data storage and analysis, knowledge discovery and computational complexities, scalability and data visualization, and information security. However, the article selection process is not referred and the studies have not been evaluated based on quality parameters.

#### Big data analytics applications

Vaishya et al. (2020) studied the main applications of AI for prevention and fighting against Coronavirus Disease 2019 (COVID-19). The authors recognized seven applications of AI for the COVID-19 pandemic: (1) detection of the disease, (2) monitor patient treatment, (3) contact tracing, (4) predicting cases and deaths, (5) drug production, (6) reduction of workloads, and (7) disease prevention. However, this paper fails to take into account the following: (1) few papers were investigated (2) the study selection process is not stated, and (2) the qualitative parameters were not provided. Furthermore, a detailed taxonomy was not presented based on AI techniques.

Finally, Pham et al. (2020) discussed the applications of AI techniques and big data to manage and analyze the huge volume of data derived from the COVID-19 disease. Five categories are considered for reviewing selected big data techniques, which include prediction of COVID-19 outbreak, tracking the spread of the virus, diagnosis and treatment, and drug discovery. Then, the related challenges of the reviewed solutions highlighted. Nevertheless, there is no clear method for article selection.

Due to the investigated studies, there are some weaknesses in the current big data analysis surveys as follows:

• Many articles did not assess the qualitative metrics for investigating the techniques.
• Some papers did not present any reasonable classification of data analytics techniques in the context of big data.
• Some papers did not clear the paper selection procedure.
• Many articles did not present entire categories of artificial intelligence techniques for reviewing big data analytics.

The reasons mentioned led us to write a survey paper on big data analysis using artificial intelligence mechanisms to overcome all of these lacks.

### Research selection method

This section provides guidelines for performing a systematic analysis for studying the big data analytics approaches. The systematic analysis procedure includes a clarification of finding the related studies in scientific databases (Charband & Navimipour, 2016). The following Research Questions (RQs) are defined and answered according to the objectives and scope of the present survey:

• RQ1: What is the taxonomy designed for big data analytics techniques?
• RQ2: Which artificial intelligence techniques are applied to big data analytics?
• RQ3: What qualitative features are assessed in artificial intelligence approaches?
• RQ4: And what are the big data analytics open issues?

**Figure 1:**
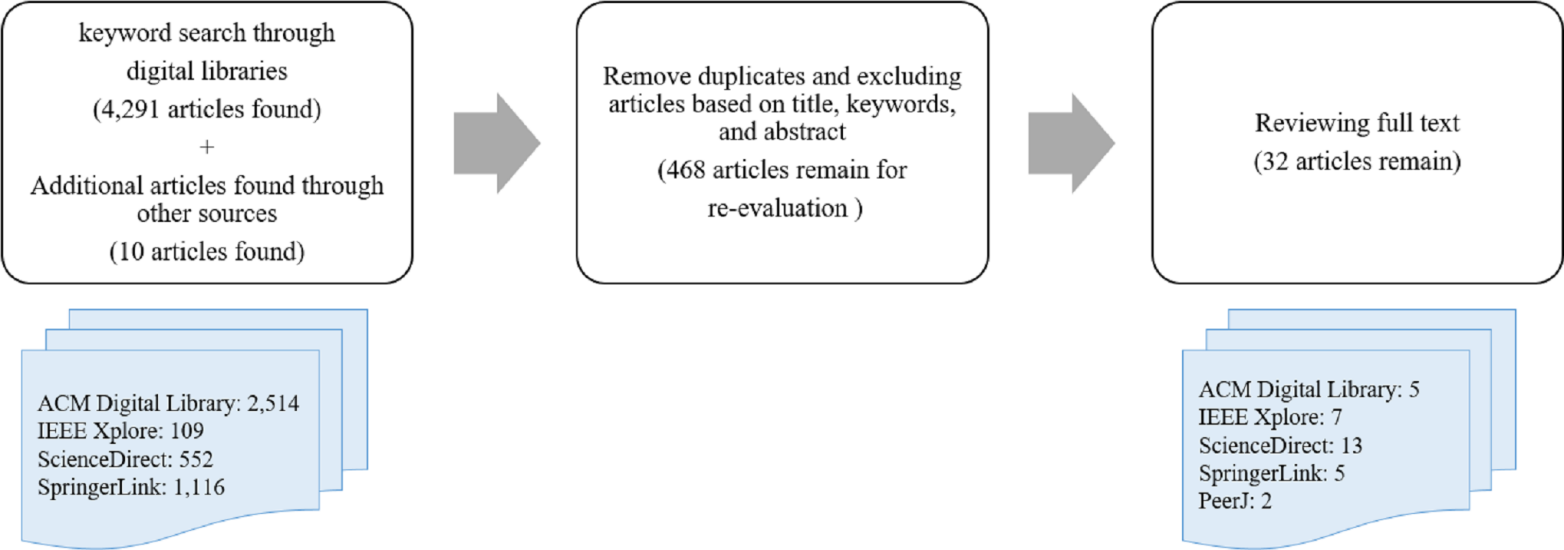
The selection process for choosing relevant studies.

After defining the research questions, some criteria are applied to select the final studies. The article selection process is shown in Fig. 1. In this systematic procedure, some popular databases such as ScienceDirect, SpringerLink, IEEE Xplore, and ACM Digital Library are used. Masters theses and doctoral dissertations, conference papers, book chapters, and non-English papers were excluded from the study. The following keywords are searched for the period 2016 to 2021 (Antonopoulos et al., 2020):

• “Artificial Intelligence” AND “Big Data Analytics”
• “Machine Learning” AND “Big Data Analytics”
• “Neural Networks” AND “Big Data Analytics”

**Figure 2:**
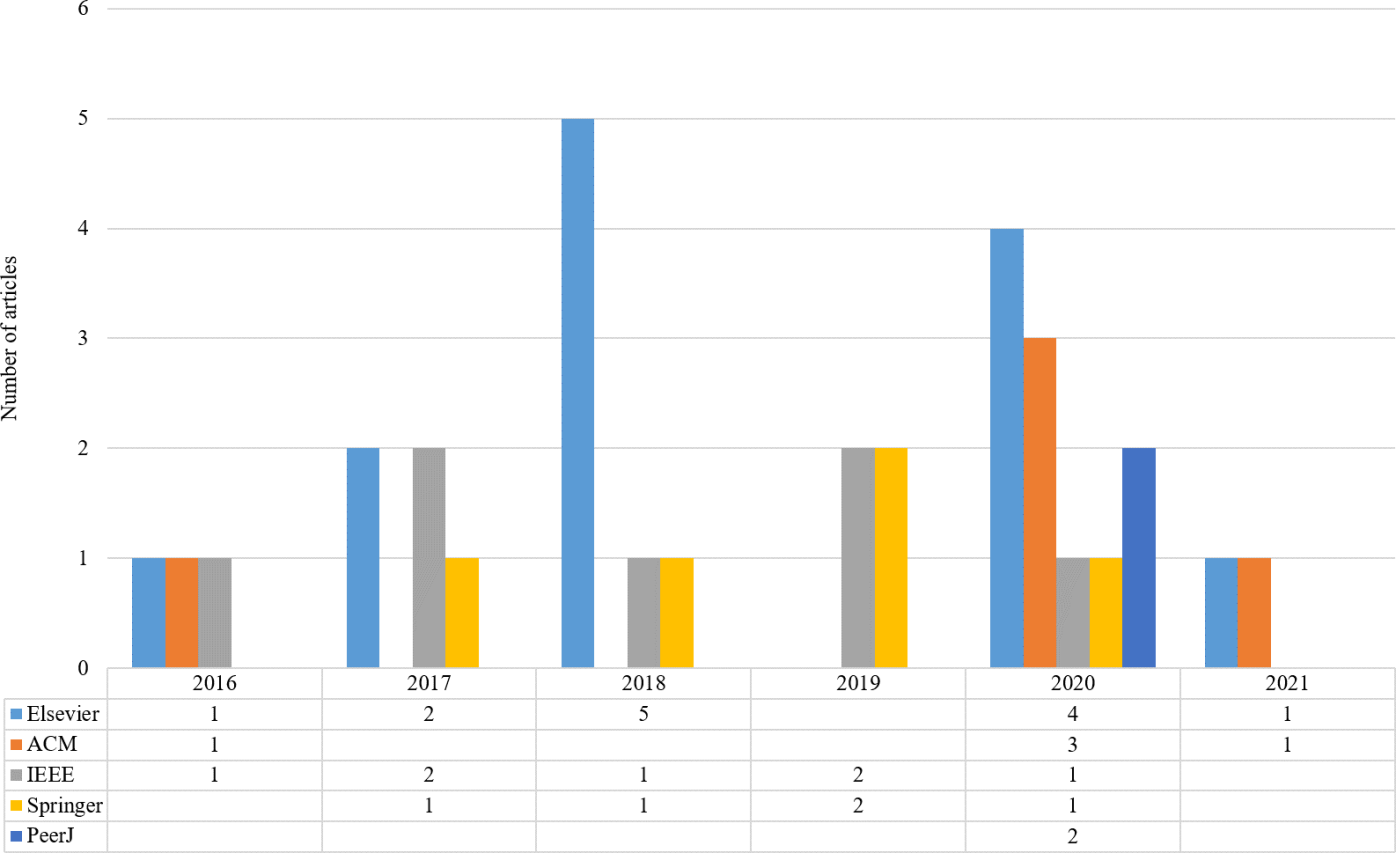
Distribution of the articles by various publishers and publication year.

Initially, 4,291 and additional 10 papers were identified through our keyword search strategy. In the next steps, duplicate records are removed and some criteria are considered for selecting high-quality studies. Titles, abstracts, and keywords were studied to select the articles for the next step. Henceforth, 468 articles remained for re-evaluation. In stage 3, a review of the text of the selected studies from the second stage was performed to confirm these studies. A total of 32 articles were identified in the last step. The distribution of the articles by various publishers and the publication year is shown in Fig. 2. As shown in Fig. 2, the highest number of articles is related to Elsevier in 2018.

### AI-driven big data analytics mechanisms

**Figure 3:**
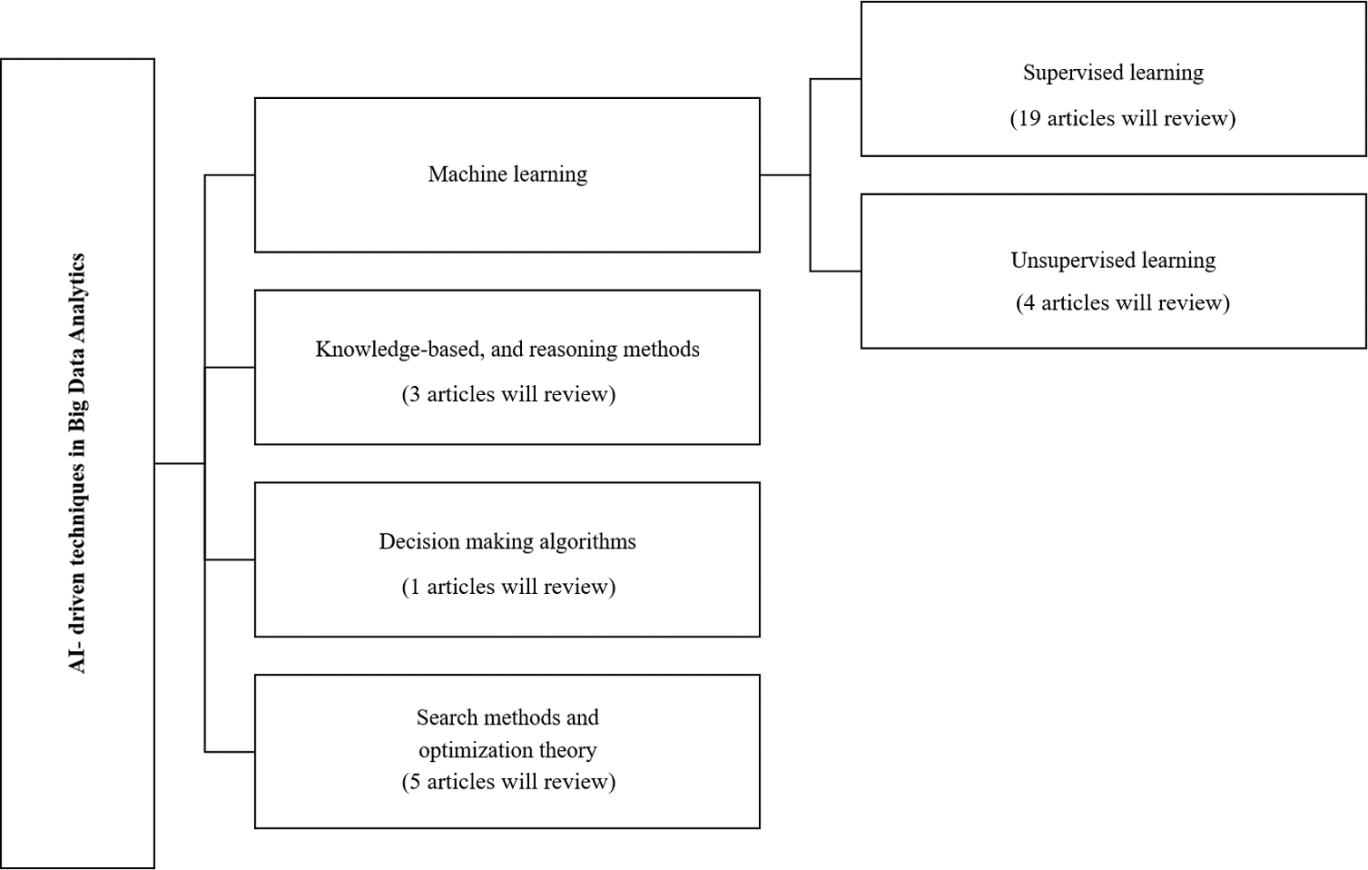
Schematic diagram of classification of AI.

Classification and review of the selected big data analysis studies are performed based on the AI subfields used in big data analytics. Figure 3 shows the taxonomy of the big data analytics techniques based on the AI subfields, and categorizes the articles investigated in this survey within those categories. The presented taxonomy has four main categories, including machine learning, knowledge-based and reasoning methods, decision-making algorithms, and search methods and optimization theory (Russell & Norvig, 2020).

Furthermore, the four most significant qualitative parameters are defined to assess each big data analysis method and recognize its benefits and drawbacks, as follows:

• **Scalability**: The mechanism’s ability to adapt to rapid changes without compromising the quality of the analysis.
• **Efficiency**: It denotes the ratio of the method to the overall time and cost need.
• **Precision**: This is detected with various parameters like data errors, and the predictive ability of algorithms.
• **Privacy**: It defines the practices which safeguard that the data is only used for its intended purpose.

The papers are overviewed and compared with mechanism goals in the last step.

### Machine learning mechanisms

Machine learning algorithms can be divided into two main classes including supervised learning and unsupervised learning. The first class needs a lot of manual effort to put the data in a proper format to learn algorithms. The unsupervised learning algorithms can discover hidden patterns in huge amounts of unlabeled data.

#### Supervised learning

**Figure 4:**
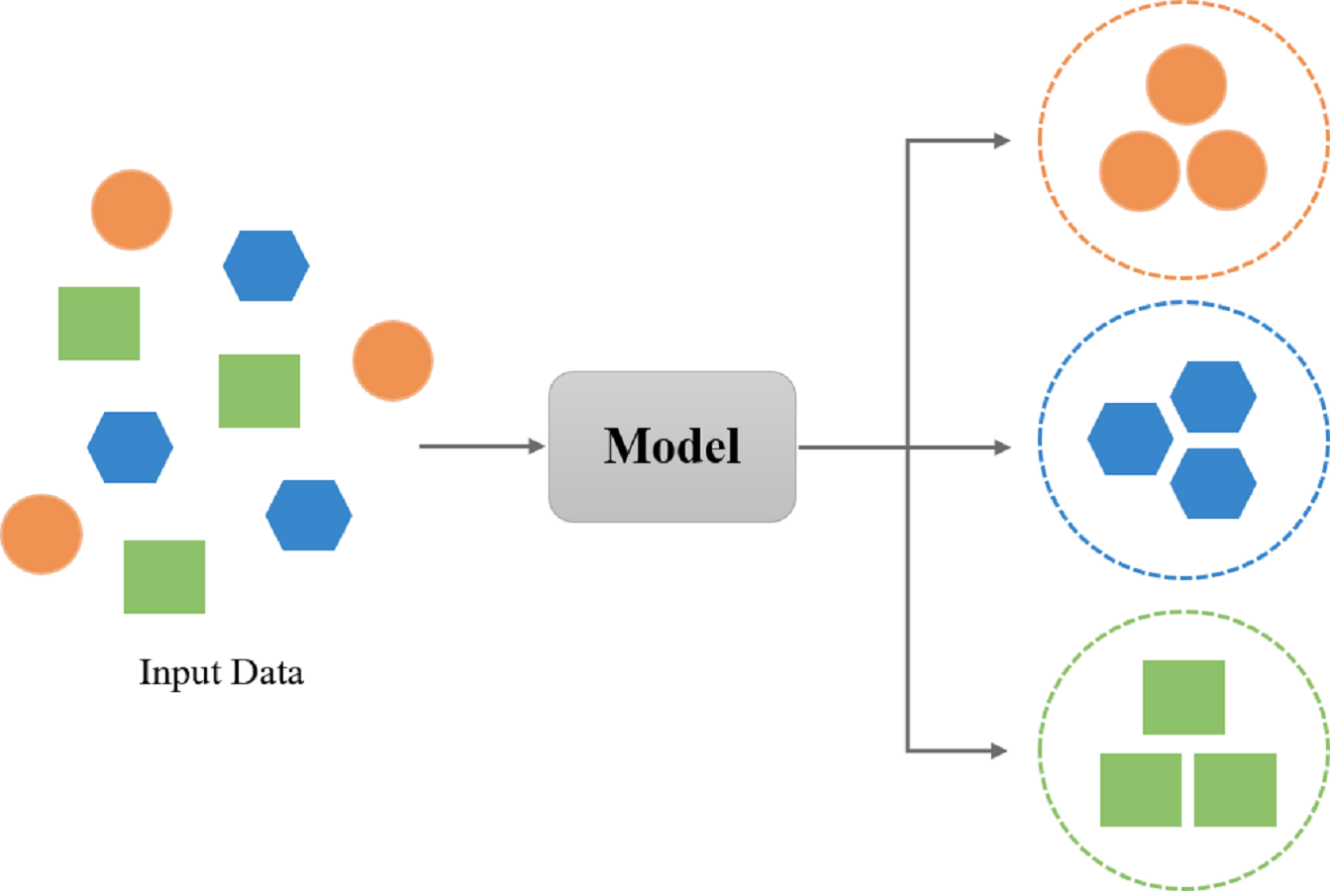
Overview of supervised learning.

The aim of a supervised learning algorithm is to forecast the right label for newly presented input data using another dataset. In this learning method, a set of inputs and outputs is presented and the relation among them is found while training the system. The main objective of supervised learning is to model the dependency between the input features and the target prediction outputs. As shown in Fig. 4, input examples are categorized into a known set of classes (Kotsiantis, Zaharakis & Pintelas, 2007).

Carcillo et al. (2018) proposed a novel platform for fraud detection named, SCAlable Real-time Fraud Finder (SCARFF). The proposed platform uses Kafka, Spark, and Cassandra big data tools along with a machine learning technique to process streaming data. The machine learning engine composed of a weighted ensemble that employs two types of classifiers based on random forest (Breiman, 2001; Rokach, 2016). It deals with imbalanced data, non-stationarity, and feedback latency. The results indicate that the efficiency, accuracy, and scalability of the presented framework is satisfactory over a big stream of transactions.

Kannan et al. (2019) presented a predictive approach on demonetization data using a support vector machine, called PAD-SVM. Preprocessing, descriptive analysis, and prescriptive analysis are three stages of the proposed PAD-SVM. Cleaning the data, handling the missing data fields, and splitting the essential data from the tweets are performed in the first stage. Identifying the most influential individual and performing analytical functionalities are two key functions of the descriptive analysis stage. Semantic analysis is also performed in the second stage. The present mindset of people and the reaction of society to the problem is predicted using predictive analysis. The authors performed a series of experiments and confirmed the performance of the proposed method in terms of execution time and classification error.

Feng et al. (2019) proposed several data mining and deep learning methods for visualization and trend prediction of criminal data. The authors discovered various interesting facts and patterns from the criminal data of San Francisco, Chicago, and Philadelphia datasets. The proposed method has lower complexity in comparison with LSTM. Based on the predictive results of the article, the superior performance of the Prophet model and Keras stateful LSTM is confirmed as compared to traditional neural networks.

Accurate and timely forecasting popularity of television programs is of great value for content providers, advertisers, and broadcast television operators. Traditional prediction models require a huge amount of samples and long training time, and the precision of predictions for programs with high peaks or severe decrease in popularity is poor. Zhu, Cheng & Wang (2017) proposed an enhanced prediction method based on trend detection. The authors used a random forest model after clustering the trends using the Dynamic Time Wrapping (DTW) algorithm and K-medoids clustering. For new programs, the GBM classifier applied to assign them to the existing trends. According to the trial outcomes, the introduced model obtains better prediction results with a combination of prediction values from the trend-specific models and classification probabilities. The results also revealed that the forecasting period is effectively reduced compared to the current forecasting methods.

Big data produced by social media is a great opportunity to extract valuable insights. With the growth of the data size, distributed deep learning models are efficient for analyzing social data. Henceforth, it is essential to improve the performance of deep learning techniques. Hammou, Lahcen & Mouline (2020) presented a novel efficient technique for sentiment analysis. The authors tried to adopt fastText with Recurrent Neural Network (RNN) variants to represent and classify textual data. Furthermore, a distributed system based on distributed machine learning has been proposed for real-time analytics. The performed trials prove that the presented method outperforms Long Short-Term Memory (LSTM), Bidirectional Long Short-Term Memory (BiLSTM), and Gated Recurrent Unit (GRU) methods in terms of classification accuracy. Also, it can handle large scale data for sentiment analysis.

Nowadays, the urban network has produced a huge amount of data. Therefore, some security challenges arise because of the private data gathering by smart devices. Tian et al. (2020) tried to discover the abnormal behavior of insiders to avoid urban big data leakage. The authors developed various deep learning methods to analyze deviations among realistic actions and the normalcy of daily activities. Abnormal activities are recognized using a Multi-Layer Perceptron (MLP) based on the computed deviations. According to the trial outcomes, the proposed method can learn the normal pattern of behaviors and identify abnormal activities with high precision.

Internet traffic is growing rapidly in the age of multimedia big data. Therefore, data processing and network overload are two key challenges in this context. Wang et al. (2016) proposed a hybrid-stream model to solve these challenges for video analysis. It contains data preprocessing, data classification, and data-load-reduction modules. A modified version of the CNN method is developed to evaluate the importance of each video frame to improve classification accuracy. The outcomes confirmed that the proposed model reduces data load, controls the video input size, and decreases the overload of the network. The outcomes also confirmed the effective reduction of processed video without compromising the quality of experience. Also, it observed that the model has a good performance for the continuous growth of large multimedia data as compared to other traditional models.

Kaur, Sharma & Mittal (2018) proposed a novel model for smart healthcare information systems using machine learning algorithms. The proposed model includes four layers. The data source layer handles heterogeneous data sources. The data storage layer manages the storage optimization process. Various techniques like indexing and normalization have been used to make optimal use of system resources. Different data security and privacy techniques such as data masking, granular control over data access, activity monitoring, dynamic encryption, and endpoint validation are used in the data security layer. Finally, machine learning methods used in the application layer for early diagnosis of the disease. Based on the trial outcomes of the article, the accuracy of the proposed model improved by using fuzzy logic and information theory.

Nair, Shetty & Shetty (2018) introduced a novel health status prediction system by applying machine learning models on big data streams. The presented system built using Apache Spark and deployed in the cloud environment. The user sends his health qualities and the system forecasts the user’s health status in real-time. A decision tree model is created from the existing healthcare data and applied to streaming data for health status prediction. The presented architecture leads to the time and cost-efficiency of the introduced system. The privacy of data is overcome by using a secondary Twitter account.

AlZubi (2020) developed new big data technologies and machine learning methods to identify diabetes disease. First, the data is gathered from a huge data set, and the MapReduce model is used to efficiently combine the small chunk of data. Then, the normalization procedure is used to eliminate the noise of the collected data. Also, an ant bee colony algorithm is applied to select the statistical features. The chosen features are trained using the SVM with a multilayer neural network. The associated neural network is applied to classify the learned features. The results revealed that the SVM neural network provides high accuracy, sensitivity, and less error rate.

Detection of COVID-19 based on the analysis of chest X-ray and Computed Tomography (CT) scans, has attracted the attention of researchers. COVID-19 medical scans analysis using machine learning algorithms provides an automated and effective diagnostic tool. El-bana, Al-Kabbany & Sharkas (2020) proposed a multi-task pipeline model based on deep neural networks for COVID-19 medical scans analysis. An Inception-v3 deep model fine-tuned using multi-modal learning in the first stage. A Convolutional Neural Network (CNN) architecture is used to identify three types of manifestations in the second stage. Transfers learning from another domain of knowledge to generate binary masks for segmenting the regions related to these manifestations are performed in the last stage. Based on the trial results, the proposed framework enhances efficiency in terms of computational time. Furthermore, the proposed system has higher accuracy compared to the recent literature.

A novel Computer-Aided Diagnosis (CAD) system called FUSI-CAD based on AI techniques has been proposed by Ragab & Attallah (2020). The proposed FUSI-CAD is based on combining several different CNN architectures with three handcrafted features including statistical features and textural analysis features that have not previously been used in coronavirus diagnosis. The results reveal that the proposed FUSI-CAD can precisely distinguish between COVID-19 and non-COVID-19 images compared to the recent related studies.

Also, a deep CNN on chest X-rays is proposed by Ahmed, Bukhari & Keshtkar (2021) to determine COVID-19. After 5-fold cross-validation on a multi-class dataset consisting of COVID-19, Viral Pneumonia, and normal X-ray images, the proposed method achieved a classification accuracy of 90.64%.

Recently, the novel coronavirus infection is threatening human health. The Internet of Things (IoT) and big data technologies play a vital role to fight against COVID-19 infection. Ahmed et al. (0000) proposed a new framework for analyzing and forecasting COVID-19 using the integration of big data analytics and IoT. The proposed framework is developed based on neural networks. According to the trial results, the proposed framework has good performance with an accuracy of 99% as compared to traditional machine learning methods.

Asencio-Cortés et al. (2018) investigated the use of regression algorithms with ensemble learning for predicting the magnitude of the earthquakes. The Apache Spark distributed processing framework along with linear regression, Gradient Boosting Machines (GBM), deep learning, and random forests machine learning models from the H2O library have been employed in this paper. The experiments demonstrate the accuracy of the tree-based methods. High levels of parallelism and scalability are the two main strengths of the introduced method. But it has low efficiency for processing large data sets.

Wang et al. (2017) developed a new model for predicting electricity prices based on a combination of some modules. To eliminate redundant features, a hybrid feature selection based on Grey Correlation Analysis (GCA) is proposed with the integration of random forest and Relief-F algorithm. A combination of the Kernel function and Principle Component Analysis (PCA) is also developed for dimensionality reduction. Furthermore, a Differential Evolution (DE) based Support Vector Machine (SVM) classifier is developed for price classification. Based on the obtained numerical results, the superior performance of the proposed technique is revealed in terms of accuracy and time efficiency.

Vu et al. (2020) used a deep learning method to capture the association between the data distribution and the quality of partitioning methods. The presented method executes in two stages including offline training and application. In the training phase, synthetic data are generated based on various distributions, divided using different partitioning techniques, and their quality is measured using different quality criteria. The data set is also summarized using histograms and skewness measures. The deep learning model trained using the data summaries and the quality metrics. The trained model applied to forecast the ideal partitioning technique given a new dataset that needs to be partitioned. The experiments revealed that the introduced method performs better than the baseline method in terms of precision in choosing the best partitioning method.

Huang et al. (2016) designed a parallel ensemble algorithm, Online Sequential Extreme Learning Machine (PEOS-ELM), based on the MapReduce distributed model for large-scale learning. The proposed PEOS-ELM algorithm supports bagging, subspace partitioning, and cross-validation to analyze incremental data. PEOS-ELM performance compared with the original Online Sequential Extreme Learning Machine (OS-ELM). Based on the results, the presented distributed algorithm can process large-scale datasets and performs well in terms of speed and accuracy.

Banchhor & Srinivasu (2020) presented a big data classification model using Cuckoo–Grey wolf based Correlative Naive Bayes classifier and MapReduce Model (CGCNBMRM). In the proposed algorithm, the Correlative Naive Bayes (CNB) classifier is enhanced by using the Cuckoo–Grey Wolf Optimization (CGWO) algorithm. CGWO is developed by a combination of Cuckoo Search (CS) and Grey Wolf Optimizer (GWO) algorithms. Henceforth, the modified CNB classifier improved by the ideal selection of the model parameters. The results proved the effectiveness of big data classification in terms of accuracy, sensitivity, and specificity.

Table 1 displays a comparison of the functional properties of the supervised-learning based big data analytics approaches. This comparison examines scalability, efficiency, precision, and privacy based on the claimed results of the investigated studies. The important factors that have increased with most of the supervised learning-based mechanisms are efficiency and precision. However, scalability and privacy have received less attention from researchers.

**Table 1:** Summary of the big data analytics in supervised learning mechanisms.

Paper	AI technique	Scalability	Efficiency	Precision	Privacy
Carcillo et al. (2018)	Ensemble classifier based on random forest algorithms	√	√	√	X
Kannan et al. (2019)	Support vector machine	X	√	√	X
Feng et al. (2019)	LSTM neural network	X	√	√	X
Zhu, Cheng & Wang (2017)	Random forests regression, and gradient boosting decision tree	X	√	√	X
Hammou, Lahcen & Mouline (2020)	Recurrent neural network	√	X	√	X
Tian et al. (2020)	LSTM neural network	X	√	√	X
Wang et al. (2016)	Convolutional neural network	√	√	√	X
Kaur, Sharma & Mittal (2018)	Various machine learning methods like naive bayes, support vector machine, and decision tree	X	X	√	√
Nair, Shetty & Shetty (2018)	Decision tree model	√	√	√	√
AlZubi (2020)	SVM-trained multilayer neural network	X	X	√	X
El-bana, Al-Kabbany & Sharkas (2020)	Convolutional neural networks with transfer learning	X	√	√	X
Ragab & Attallah (2020)	Convolutional neural network with transfer learning	X	X	√	X
Ahmed, Bukhari & Keshtkar (2021)	Convolutional neural network	X	X	√	X
Ahmed et al. ()	Neural network	X	X	√	X
Asencio-Cortés et al. (2018)	Regression algorithms with ensemble learning	√	X	√	X
Wang et al. (2017)	Differential evolution SVM classifier	X	√	√	X
Vu et al. (2020)	Deep learning	X	X	√	X
Huang et al. (2016)	Ensemble of extreme learning machines	√	√	√	X
Banchhor & Srinivasu (2020)	Naive bayes classifier improved by using CGWO	X	X	√	X

#### Unsupervised learning

**Figure 5:**
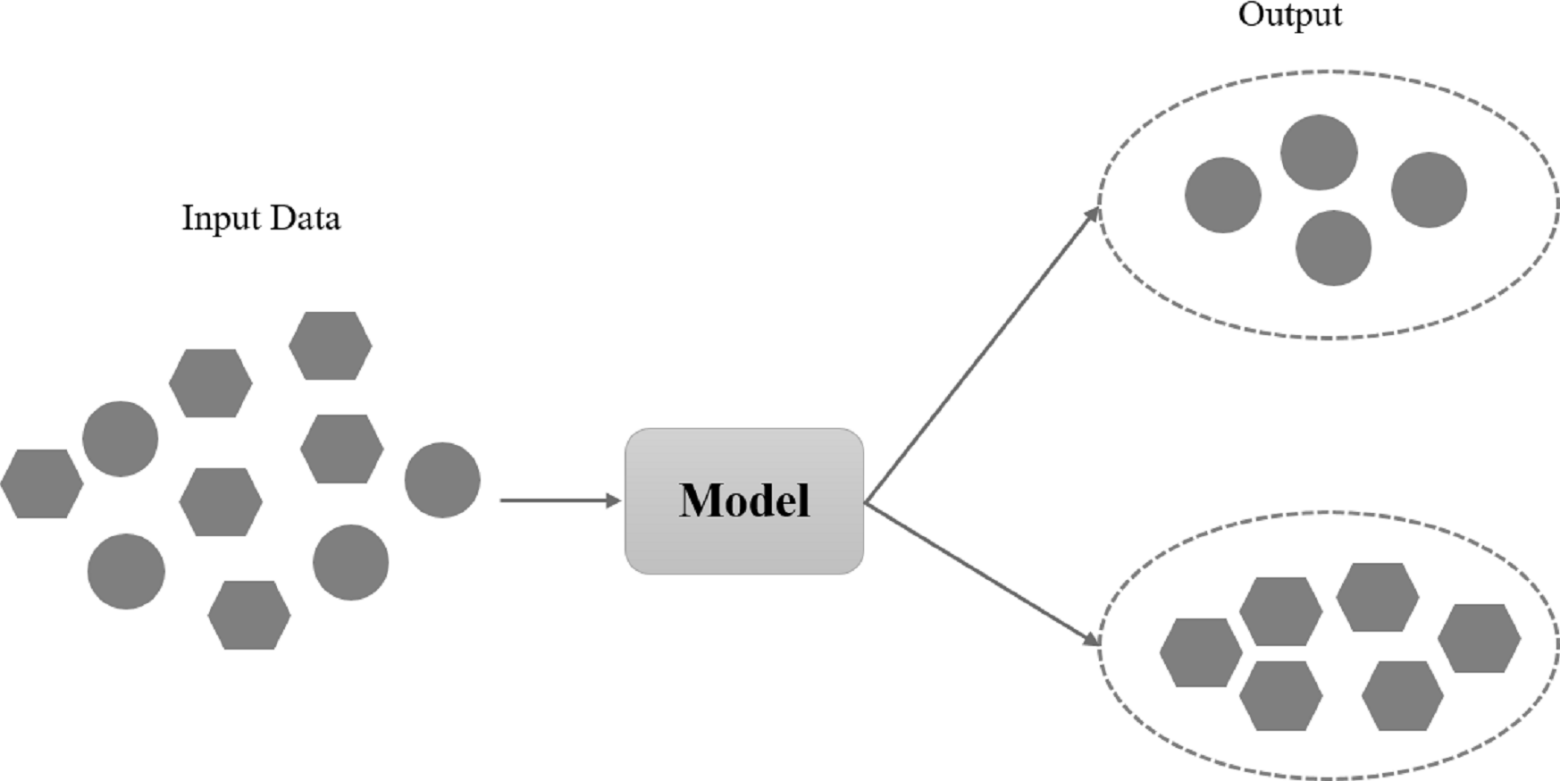
Overview of unsupervised learning (Banchhor & Srinivasu, 2020).

Unsupervised learning is used for input data without the corresponding output variable. These algorithms detect hidden patterns in the data. Clustering is one of the major types of unsupervised algorithms. As shown in Fig. 5, inherent groups in input objects are discovered based on the underlying patterns (Bengio, Courville & Vincent, 2012).

Ianni et al. (2020) introduced a parallel version of CLUBS ^+^centroid-based clustering algorithm, named CLUBS-P, for efficient centroid-based clustering. The presented unsupervised algorithm provides high-quality clusters of data around the cluster centroid. The authors examined the performance of the proposed algorithm against the performance of the parallel k-means clustering. The results revealed that the algorithm can achieve high accuracy and high scalability.

Wang, Tsai & Ciou (2020) proposed a hybrid model based on the Recency, Frequency, and Monetary (RFM) model, k-means clustering, Naive Bayes algorithm, and linked Bloom filters to analyze customer data and obtain intelligent strategies. The authors performed some experiments and demonstrated the benefits of big data analytics for marketing strategies and forecasting potential customer demands. Also, linked Bloom filters can store inactive data more efficiently for future use.

Ip et al. (2018) performed an overview of big data and machine learning techniques in the field of crop protection. Furthermore, the capability of utilizing Markov Random Fields (MRF) which considers the spatial component among neighboring sites to model herbicide resistance of ryegrass is examined. The trial results demonstrated the performance of the proposed approach.

Pulgar-Rubio et al. (2017) introduced a novel method, named MEFASD-BD, for subgroup discovery. It is the first big data approximation in evolutionary fuzzy systems for subgroup discovery. MEFASD-BD is implemented based on the MapReduce model under Apache Spark. In this paradigm, the quality of the subgroups obtained for each map is analyzed according to the main dataset to enhance the quality of the subgroups. The presented method can efficiently process high dimensional datasets. The trial outcomes of the study revealed a significant reduction in execution time while maintaining the values in the standard quality.

Table 2 shows the summary of the reviewed techniques as well as their main benefits and drawbacks. The authors focused on increasing the accuracy as the main parameter in all the unsupervised learning-based mechanisms. However, scalability, efficiency, and privacy parameters have attracted lower attention.

**Table 2:** Summary of the big data analytics in unsupervised learning mechanisms.

Paper	AI technique	Scalability	Efficiency	Precision	Privacy
Ianni et al. (2020)	Centroid-based clustering	√	√	√	X
Wang, Tsai & Ciou (2020)	RFM, K-means, Naïve Bayes, Bloom filters	X	√	√	X
Ip et al. (2018)	Markov random fields	√	X	√	X
Pulgar-Rubio et al. (2017)	Multi-objective evolutionary fuzzy systems	√	√	√	X

### Search methods and optimization theory

The search-based methods can be used to find the ideal solutions for a problem. In search-based optimization, ideal decisions made based on some objectives within the given constraints. The search space in a big data environment becomes larger. Therefore, powerful search algorithms need to be developed for large-scale optimization problems (Azhir et al., 2021). The selected methods regarding search-based methods and optimization theory are described in the following.

Alkurd, Abualhaol & Yanikomeroglu (2020) proposed the application of AI, big data analytics, and real-time non-intrusive feedback to personalize wireless networks. The authors proposed a user satisfaction model to enable user feedback measurement. An evolutionary multi-objective formulation optimizes the provided Quality of Service (QoS) and user satisfaction simultaneously. The results proved that personalization enables efficient optimization of network resources. Therefore, user satisfaction and a certain level of revenue in the form of saved resources are achieved.

The data generated from the IoT environments should be processed by analytical applications. However, considering various issues like data size, velocity, and locality, the current infrastructures cannot allocate enough resources to tasks of an application efficiently. Ding et al. (2020) proposed two task allocation methods based on Particle Swarm Optimization (PSO) to enhance resource utilization with an auto-scaling guarantee for batch and stream processing. Various experiments are performed and revealed that the proposed method can increase the efficiency of resource utilization by effectively supporting the offload.

Optimizing the performance of transport protocols is a challenging task for transmitting big data over dedicated channels in High-Performance Networks (HPNs). Yun et al. (2019) proposed ProbData, PRofiling Optimization Based DAta Transfer Advisor, to adjust the number of parallel streams and the buffer size for Transmission Control Protocol (TCP) transmission using stochastic approximation. ProbData used the Simultaneous Perturbation Stochastic Approximation method to recognize the ideal transmission configurations for TCP and UDP-based transport methods. The performance of ProbData is assessed using real-life performance measurements and physical connections in current HPNs. Based on the results, the proposed method can significantly reduce the profiling overhead while achieving good performance.

With the growth of global services, the need for big data analytics in multiple Data Centers (DCs) located in different regions increases. Recent attempts to analyze geo-distributed big data cannot guarantee a predictable job completion time and lead to excessive network traffic over the inter-DC. Li et al. (2016) minimized inter-DC traffic produced by MapReduce jobs by directing geo-distributed big data while predicting job completion time. The authors formulated an optimization problem using the movement of input data and the placement of tasks. Also, the chance-constrained optimization method is applied to guarantee the predictable job completion time. Therefore, the MapReduce job can most likely be performed at a predetermined time. Several simulations have been performed using real traces produced by a series of queries on Hive. According to the trials, the proposed method reduces the inter-DC traffic compared with centralized processing by gathering all data in a single data center.

Managing and evaluating a large set of criteria is challenging in facility layout design problems. Tayal & Singh (2018) proposed a framework by integrating big data analytics and a hybrid meta-heuristic method to design an efficient facility layout over multi-period stochastic demand. First, the factors affecting the design of the facility layout are recognized. Then, using big data analysis, a reduced set of factors is obtained. The reduced set is used to model a weighted aggregate objective for the Multi-Objective Stochastic Dynamic Facility Layout Problem (MO-SDFLP). A combination of Firefly (FA) and Chaos Simulated Annealing (CSA) is applied to solve the MO-SDFLP.

Table 3 shows a comparison of the most important strengths and weaknesses of the discussed mechanisms. According to the results of the reviewed articles, search-based algorithms have high efficiency and achieve high precision results. However, these algorithms are not suitable for large-scale data.

**Table 3:** Summary of the big data analytics in search methods and optimization theory.

Paper	AI technique	Scalability	Efficiency	Precision	Privacy
Alkurd, Abualhaol & Yanikomeroglu (2020)	Evolutionary multi-objective algorithm	X	√	X	X
Ding et al. (2020)	Particle Swarm Optimization (PSO)	√	√	√	X
Yun et al. (2019)	Simultaneous perturbation stochastic approximation	X	√	√	X
Li et al. (2016)	Chance-constrained optimization	X	√	X	X
Tayal & Singh (2018)	Meta-heuristic approach based on firefly and chaotic simulated annealing	X	√	X	X

### Knowledge-based and reasoning

Knowledge-based and reasoning is one of the major fields of AI. A reasoning system can perform better than a human expert using its knowledge base within a specified domain. Three selected knowledge-based mechanisms are discussed in this section.

Recently, various classifiers have been developed to classify big data. Extended Belief Rule Base (EBRB) systems have shown their capability for big data and multiclass issues. However, time complexity and computing efficiency are two key challenges of BRB methods. Yang et al. (2018) proposed three improvements of EBRB systems to improve the time complexity and computing efficiency for multiclass classification in large data. The proposed method is based on the approach of skipping the rule weight computation, an evidential reasoning algorithm, and a rule reduction method based on domain division. Moreover, parallel rule generation and inference schemes of the proposed classifier are implemented under Apache Spark. Based on the results, the EBRB can obtain good accuracy and have better time complexity and computing efficiency than some popular classifiers.

Recently, context-aware computing has received increasing attention in the IoT and pervasive computing. Context acquisition, context modeling, and context-aware reasoning are three major steps of this method. Although, the development of context-aware applications for reasoning on resource-bounded mobile devices is challenging. Rakib & Uddin (2019) presented a context-aware framework with a lightweight rule engine and a wide range of user preferences to decrease the number of rules while inferring personalized contexts. The authors confirmed that associated rules can be reduced in order to enhance the inference engine efficiency in terms of accuracy, execution speed, total execution time, and execution cost.

Araújo & Pestana (2017) proposed a novel solution to increase employee’s motivation and encouraging them to be more active. It is performed by automatically detecting stressful situations and offering recommendations when identifying a stressful pattern. Two notions of workplace well-being (i.e., physical and social) are aggregated with gamification methods to analyze how it can aid employees to obtain the soft and hard skills to enhance their curriculum.

Table 4 shows a comparison of the most significant benefits and drawbacks of the discussed mechanisms. Generally, the primary drawbacks of knowledge-based and reasoning mechanisms are the problems encountered during knowledge acquisition, as well as adaptability. Besides, these methods cannot be used for large quantities of data.

**Table 4:** Summary of the big data analytics in knowledge-based and reasoning.

Paper	AI technique	Scalability	Efficiency	Precision	Privacy
Yang et al. (2018)	Belief rule base systems	√	√	√	X
Rakib & Uddin (2019)	Rule-based reasoning	X	√	√	X
Araújo & Pestana (2017)	Gamification rules	X	X	√	X

### Decision making algorithms

The aim of decision algorithms is to maximize the expected utility. In these algorithms, the desirability of a state is calculated using a utility function. The agent decides with the aim of maximizing the utility function. The selected decision making-based mechanism is discussed in the following.

Big data analytics applications need to be re-deployed when changes are occurred in the Cloud at runtime. Lu et al. (2017) presented a decision-making solution for selecting the most appropriate deployment for big data analytics applications. First, a new language called DepPolicy is presented to specify runtime deployment information as policies. Then, MiniZinc is developed to model the deployment decision problem as a constraint programming model. Then, a decision-making algorithm is introduced to make various deployment decisions based on total utility maximization while satisfying all given constraints. Finally, a decision making middleware, called DepWare, is applied to deploy the application in the Cloud. The obtained result confirmed the functional correctness, performance and scalability of the proposed method.

**Table 5:** Summary of the big data analytics in decision making algorithms.

Paper	AI technique	Scalability	Efficiency	Precision	Privacy
Lu et al. (2017)	Constraint programming-based decision making model	√	√	√	X

Table 5 shows the most significant benefits and drawbacks of the discussed mechanism.

## Results and Comparisons

The selected AI-driven big data analysis mechanisms have been surveyed in the previous section. We described the most important AI-driven big data analysis techniques until 2021. As mentioned in the previous sections, machine learning, knowledge-based and reasoning methods, decision-making algorithms, and search methods and optimization theory are four main categories of big data analytics techniques. The main achievements of these techniques are: first, AI drives down the time taken to perform big data analytics. Repetitive tasks can be done with the help of machine intelligence. Reducing the error and enhancing the degree of precision is another advantage of AI-driven big data analytics.

**Figure 6:**
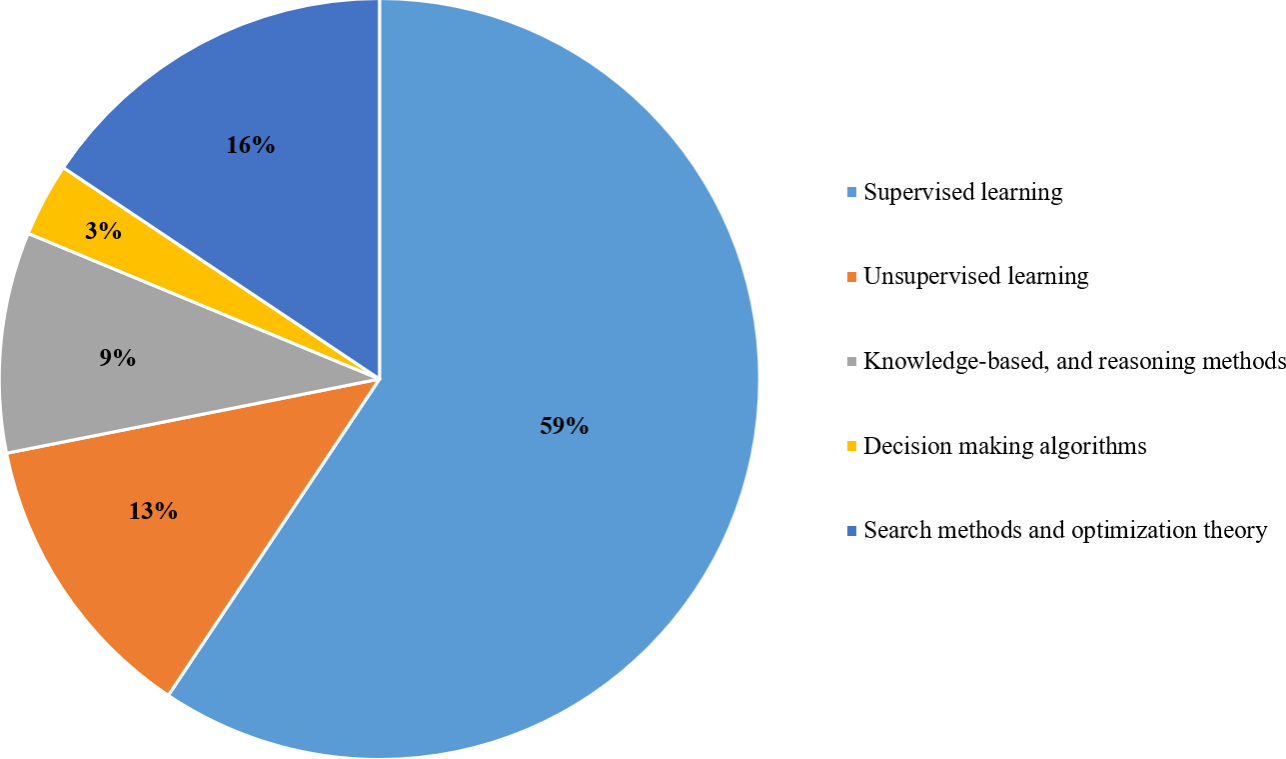
Various types of AI techniques used in the selected articles.

**Figure 7:**
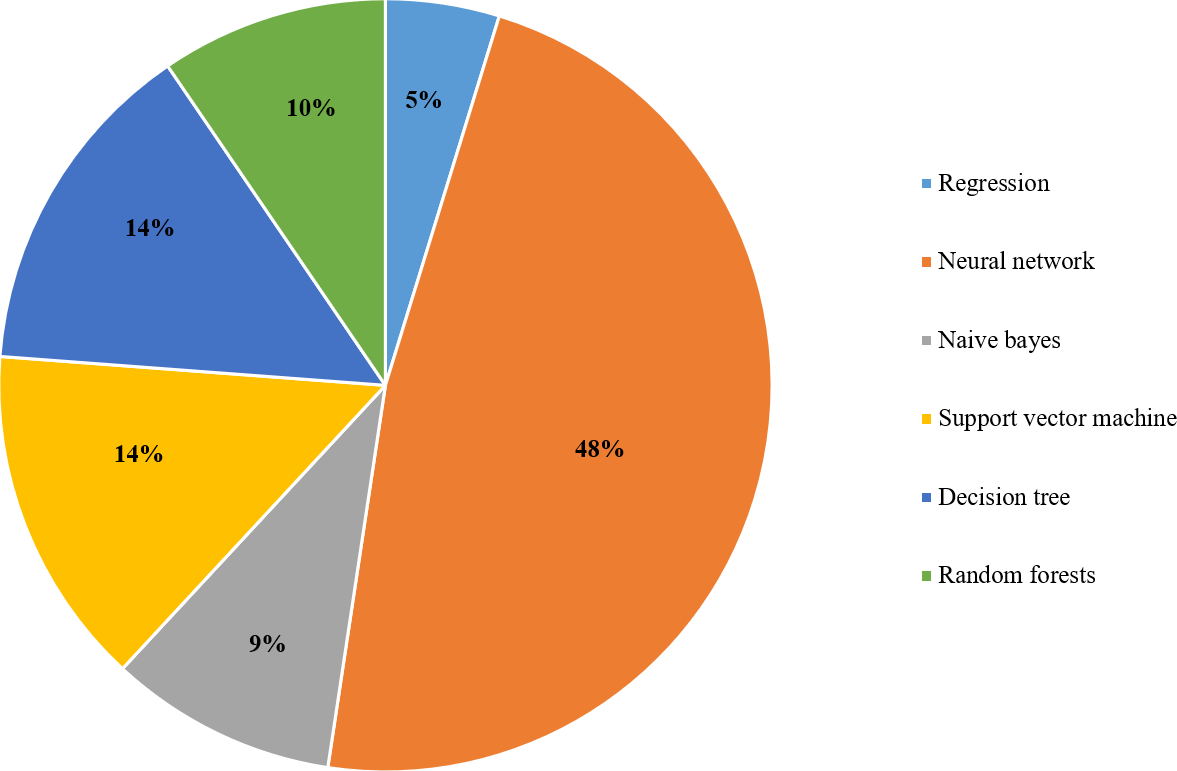
The supervised learning algorithms used for big data analytics in the selected articles.

As shown in Fig. 6, the popular technique that researchers use to analyze big data is supervised learning with 59%. Relevant techniques include regression, ensemble classifier, naive bayes, decision tree, random forest, support vector machine, and neural network. Also, Fig. 7 displays the popularity of the various supervised learning techniques in big data analytics, which clearly shows that neural networks, SVM, and decision trees are the most popular ones.

Also, we evaluate the parameters which have an impact on the big data analysis based on artificial intelligence approaches. The main features of the studied big data analysis techniques, which include scalability, efficiency, precision, and privacy are provided in Tables 1–5. Based on the claimed results of the investigated articles, the machine learning-based mechanisms focus on improving the accuracy of big data analytics. However, the machine learning-based mechanisms have high complexity and overhead compared with other mechanisms. The search-based methods focus on optimization and efficiency. Also, it suffers from low scalability for large scale data. Also, knowledge-based and reasoning mechanisms have high accuracy. Finally, the investigated decision making algorithm guarantee the scalability, efficiency, and precision metrics.

**Figure 8:**
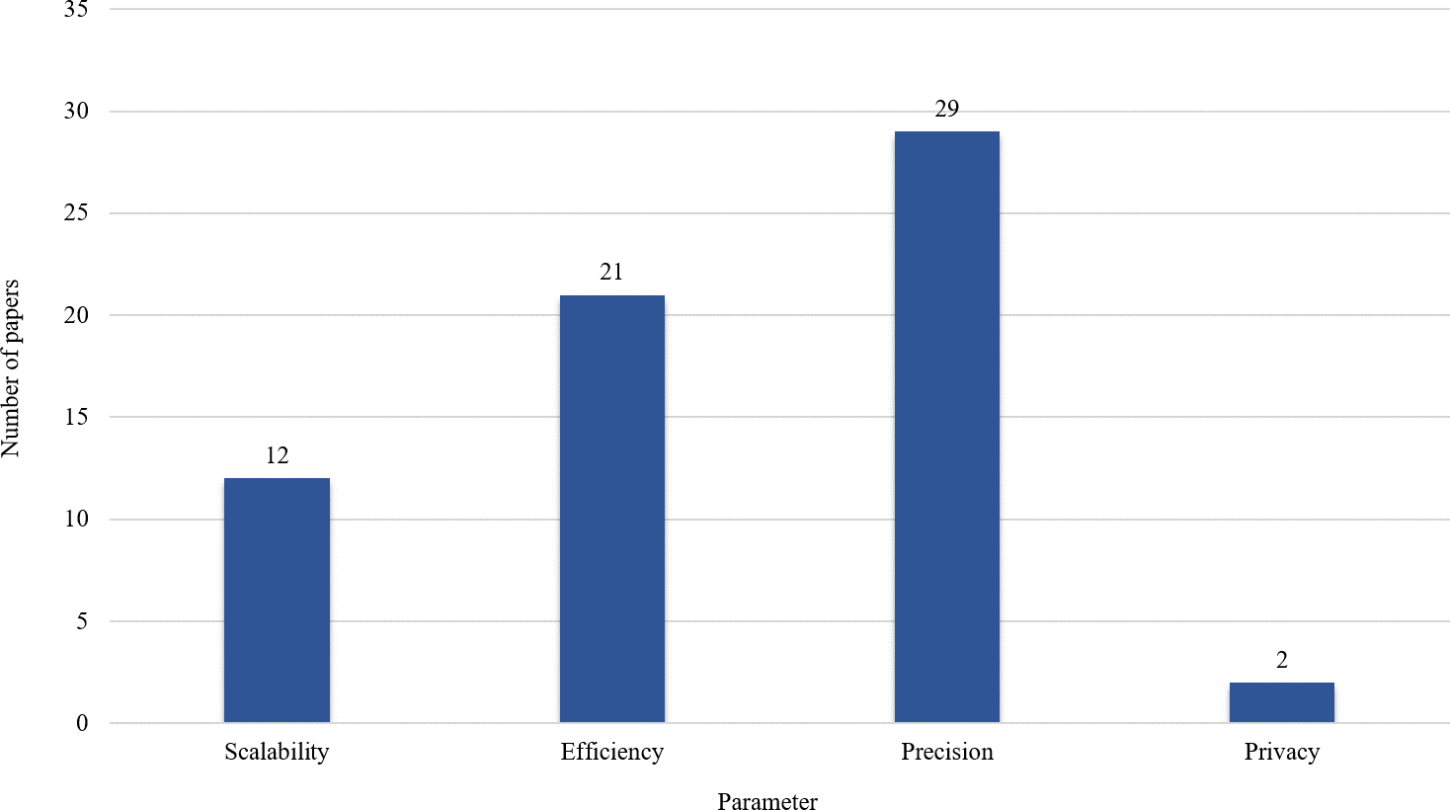
Parameters considered in the selected articles.

Figure 8 shows the outcomes of the provided results in Tables 1–5. These outcomes reveal that precision and efficiency are at the center of attention. Scalability is an important parameter that should be considered more in the future. Also, privacy is another challenging research area that is not considered in many big data analysis techniques.

### Open issues and challenges

This part offers some challenges for big data analytics using AI techniques from various perspectives: (1) Fog computing; (2) Processing huge quantities of data; (3) Security; (4) Qualitative parameters and metrics; (5) and Data quality.

• **Fog computing**. The IoT architecture produces large quantities of data that need to be analyzed in real-time. Fog computing is a technology that employs edge devices to provide a considerable quantity of computation, storage, and communication locally. It is recommended that more research should be done for Big IoT data analytics by fog computing structure.
• **Processing huge quantities of data**. Big data is produced from numerous, distributed, and heterogeneous sources and has different features such as high-speed, huge volume, heterogeneity of data formats, incomplete, and inconsistent. Processing an enormous amount of unstructured, inconsistent, incomplete, and imprecise data by computing machines is a challenging task. This data cannot be stored and processed by traditional data processing methods. Various artificial intelligence techniques must be implemented to analyze such huge quantities of data in real-time. Henceforth, the efficiency and scalability of current analytics algorithms being applied to big data must be investigated and improved.
• **Security**. Without a secure way to handle the collected big data from various systems and environments, big data analytics cannot be a reliable system. The security issues of big data analytics should be handled in various fields such as protecting IoT devices from attacks, secure AI techniques, and secure communication with external systems. To the best of our knowledge, there are few studies focusing on the security issues of big data analytics. Investigating security challenges and measures is an interesting research line in the future.
• **Qualitative parameters and metrics**. As studied in this paper, various AI techniques applied to different datasets. The authors used different quality attributes for validation of the presented techniques. Although, the study of big data analytics on the same real-world datasets, with the same techniques and the same experimental infrastructure and their assessment by considering the various quality attributes is very interesting.
• **Data quality**. Big data includes huge volumes of semi-structured and unstructured data, like JSON and text documents. Moreover, more research with a focus on data quality problems for unstructured, and semi-structured data formats is needed.

## Conclusion

The state of the art mechanisms in the field of big data analytics is surveyed in this article. According to the performed study, we introduced a taxonomy for AI-driven big data analytics mechanisms. The selected 32 articles are investigated in four main categories including machine learning, knowledge-based and reasoning methods, decision-making algorithms, and search methods and optimization theory. The advantages and disadvantages of each of these mechanisms have been investigated. The machine learning-based mechanisms use a learning method to adapt the automated decisions. Efficiency and precision as the major factors are improved in most of the machine learning-based mechanisms. However, the use of incomplete and inconsistent data may produce incorrect results. The search-based optimization methods used various objective functions to find an optimal solution from a number of alternative solutions. These methods have high efficiency and high precision. Although, these methods are not scalable enough. The knowledge-based and reasoning mechanisms improve the analytics quality using the knowledge base. The major advantage of knowledge-based mechanisms is their relative simplicity of development. Although coverage for different scenarios is lower, whatever scenarios are covered by these mechanisms will provide high accuracy. In decision making algorithms, a decision making problem is modeled as a constraint programming problem and the desirable decision is made using a utility function maximization. These mechanisms have good performance in terms of scalability, efficiency, and precision. Furthermore, this survey introduces some interesting lines for future research.

The data gathered in this paper aid to explain the state-of-the-art in the field of big data analysis. This survey tries to perform a detailed systematic study but also has some limitations. It fails to study big data analysis techniques that are available in different sources. Furthermore, the articles which are not in the context of big data are not entirely investigated. Despite this, the results will help researchers to develop more effective big data analysis methods in big data environments.

## Additional Information and Declarations

### Data Availability

The following information was supplied regarding data availability: This article is a literature review.
